# Association of inflammation and protein carbamylation in patients with COVID-19

**DOI:** 10.3389/fmed.2025.1561670

**Published:** 2025-04-02

**Authors:** Jolanta Smykiewicz, Ryszard Tomasiuk, Roman Cemaga, Jakub Buczkowski, Mateusz Maciejczyk

**Affiliations:** ^1^Dr. Tytus Chałubiński Specialist Hospital in Radom, Radom, Poland; ^2^Faculty of Medical Sciences and Health Sciences, Casimir Pulaski University of Radom, Radom, Poland; ^3^Students’ Scientific Club “Biochemistry of Civilization Diseases” at the Department of Hygiene, Epidemiology and Ergonomics, Medical University of Białystok, Białystok, Poland; ^4^Department of Hygiene, Epidemiology and Ergonomics, Medical University of Białystok, Białystok, Poland

**Keywords:** protein carbamylation, carbamyl-lysine, COVID-19, biomarkers, CBL

## Abstract

**Introduction:**

Carbamylation involves the non-enzymatic binding of isocyanic acid to the amino groups of proteins, making it associated with many pathological conditions, including inflammation, aging, arteriosclerosis, and renal failure. However, there are no data on protein carbamylation in patients with COVID-19. Our study is the first to evaluate the association between blood inflammation and protein carbamylation in patients who died from COVID-19 compared to COVID-19 survivors.

**Methods:**

The study included 50 patients admitted to Dr. Tytus Chałubiński Specialist Hospital in Radom, Poland. Twenty-five of them were COVID-19 survivors (15 men, 10 women), and 25 were COVID-19 deceased patients (15 men, 10 women). The number of subjects was based on a pilot study assuming a significance level of 0.05 and a test power of 0.8. Plasma/serum samples were assayed for carbamyl-lysine (CBL) and inflammatory biomarkers (CRP, procalcitonin, D-dimer, IL-6, and WBC). The concentration of CBL was measured using an enzyme-linked immunosorbent assay (ELISA). Statistical analysis was performed using the Mann-Whitney U test and Spearman rank correlation. Receiver Operating Characteristic (ROC) analysis was used to assess the diagnostic utility of serum CBL.

**Results:**

Serum CBL levels were significantly higher in patients who died from COVID-19 compared to COVID-19 survivors (*p* = 0.0011). There was a positive correlation of serum CBL with IL-6, D-dimer, and WBC. Serum CBL levels >101 ng/mL, with moderate sensitivity and specificity, differentiate COVID-19 deceased from recovered patients (area under the curve 0.76).

**Discussion:**

In conclusion, COVID-19 is associated with excessive protein carbamylation. Inflammation may be a source of higher CBL production in COVID-19. A thorough understanding of the consequences of increased protein carbamylation may clarify the consequences of COVID-19 complications.

## Introduction

As of December 2024, the World Health Organization (WHO) has reported more than 776.8 million cases of coronavirus disease (COVID-19) and more than 7 million deaths (1). The innate immune response to severe acute respiratory syndrome coronavirus 2 (SARS-CoV-2) is inflammation. Upon entering cells, the virus activates the pattern recognition receptor (PRR) to detect pathogen-associated molecular patterns (PAMPs) (2). Under such conditions, host immune cells produce pro-inflammatory cytokines [tumor necrosis factor α (TNF-α); interleukin-1α (IL-1α); interleukin-6 (IL-6)] and chemokines [C-C motif chemokine ligand 2, (CCL2) (2) and C-C motif chemokine ligand 8 (CCL8)]. A cytokine storm is an immune dysregulation characterized by systemic inflammation (hypercytokinemia) and multi-organ dysfunction. Many patients with COVID-19 have respiratory symptoms, including cough and tachypnoea, which can lead to acute respiratory distress syndrome (ARDS) (3). Although most of the COVID-19-related deaths happened at the beginning of the pandemic, SARS-CoV-2 occurs with no apparent seasonality and causes severe acute illness and post-COVID-19 conditions (1). Therefore, a thorough understanding of the causes of COVID-19 and the development of new therapeutic approaches is still a public health concern.

Proteins undergo numerous post-translational modifications, including prolyl and lysyl residues hydroxylation, deformylation of the N-terminal methionine, or generation of disulfide bridges/protein cross-links (4, 5). The modified protein gains unique properties or loses biological activity (6). The most well-known modification of proteins is non-enzymatic glycation involving the coupling of an aldehyde/ketone group of a sugar to the free amino group of a protein, leading to the formation of advanced glycation end products (AGE) (7). AGE binding to a specific receptor (RAGE) activates an inflammatory cascade, including the release of interleukins and cytokines, overproduction of reactive oxygen species (ROS), and disruption of apoptosis and autophagy (7, 8). The involvement of AGE/RAGE signaling has been proven in the course of numerous metabolic (9), cardiovascular (10), neurodegenerative (11), and cancer complications (12). Interestingly, RAGE is expressed mainly by type 2 epithelial cells in the alveolar sac, which plays a key role in COVID-19 lung inflammation and damage (13). Although the direct role of AGE in COVID-19 severity has not yet been clarified, there is a more significant correlation between AGE level and COVID-19 severity (13–15). However, little is known about other protein modifications in COVID-19. In19. In the available literature, we found no data on protein carbamylation in patients with COVID-19. Carbamylation involves the non-enzymatic binding of isocyanic acid to amino groups of proteins, especially under inflammatory conditions (16). Carbamylation alters proteins’ secondary and tertiary structure, affecting their biological activity and accelerated aging (16, 17). The main product of blood protein carbamylation is carbamyl-lysine (CBL), commonly known as homocitrulline (16). The association of protein carbamylation in patients with chronic renal failure has been demonstrated (18, 19) to date. It has also been shown that lipoprotein carbamylation catalyzed by myeloperoxidase (MPO) facilitates many atherosclerotic activities and, thus, may be a mechanism linking inflammation and coronary artery disease (20). However, little is known about the involvement of CBL in the pathogenesis of other inflammatory diseases. Given the complexity of COVID-19 and the interdependence of protein glycation and carbamylation, it is reasonable to know the role of CBL in the pathogenesis of COVID-19. Our study is the first to evaluate the association between blood inflammation and protein carbamylation in patients who died from COVID-19 compared to COVID-19 survivors.

## Materials and methods

### Study subjects

The study was performed according to the World Medical Association (WMA) Declaration of Helsinki. The Medical Chamber of Gdansk, Poland (KB-29/21) granted Institutional Ethics Clearance (IEC). Each person provided a signed informed consent form.

The study included 50 patients admitted to Dr. Tytus Chałubiński Specialist Hospital in Radom, Poland due to COVID-19. Twenty-five of them were COVID-19 survivors (15 men, 10 women), and 25 were COVID-19 deceased patients (15 men, 10 women). COVID-19 was confirmed by a positive test for SARS-CoV-2 genetic material in nasopharyngeal swabs by PCR assay (VIASURE SARS-CoV-2 (N1 + N2) Real-Time PCR Detection Kit for BD MAX; Becton Dickinson & Co., BD Diagnostic Systems, 7 Loveton Circle, MD, USA). Exclusion criteria in both groups were the presence of chronic inflammatory diseases such as acute and chronic kidney disease, rheumatoid arthritis, systemic scleroderma, multiple sclerosis, non-alcoholic fatty liver disease, thyroiditis, psoriasis, type 1 diabetes, and cancer.

Blood samples were collected at the last blood measurement prior to discharge from the hospital in case of surviving patients or the last blood measurement before death for patients who died from COVID-19. Blood samples were collected into tubes with ethylenediaminetetraacetate tripotassium (K3-EDTA; S-Monovette^<*reg*>(</reg>^ EDTA K3E, Sarstedt) and clotting activator (S-Monovette^<*reg*>(</reg>^ Serum Gel CAT; Sarstedt) and centrifuged at 4,000 rpm for 10 min at 4°C to separate plasma or serum. The supernatant fluid was preserved for the study and frozen at −80°C until the determinations were made.

Complete blood count (CBC), including platelets (PLT), white blood cells (WBC), and red blood cells (RBC), along with RBC parameters such as mean corpuscular hemoglobin concentration (MCHC), mean corpuscular volume (MCV), hematocrit (HCT), and hemoglobin (HGB), was performed automatically using a Sysmex XN-550 (21). Sysmex XN-550 is a multiparameter hematology analyzer with the ability to target low sample volumes.

### Blood inflammation

Procalcitonin (PCT) concentration was measured *in vitro* using the Elecsys BRAHMS PCT serum assay (22) through a three-step process. The first step involved incubation with sample antigen, biotinylated PCT-specific monoclonal antibodies, and PCT-specific monoclonal antibodies conjugated with a ruthenium complex. Next, labeled microparticles were added to streptavidin to bind the complex to the solid phase using the affinity of biotin and streptavidin. In the final step, the reaction mixture was transferred to the measuring chamber, where a magnet attracted the microparticles to the electrode surface. The unbound particles were processed using the ProCell/ProCell M method. Then, a photomultiplier was used to measure electrochemiluminescence and photon emission induced by the applied voltage. The results were quantified by reference to the calibration curve.

D-dimer’ levels were measured via photometric Dia-D-DIMER (23), an immunoturbidimetric test reinforced with latex particles. In this method, latex particles are additionally coated with antibodies directed to D-dimer. An antigen-antibody photometric reaction determines the concentration of D-dimer.

IL-6 concentration was measured in serum collected from separation gel tubes. The procedure followed the next steps: first incubation of 30 μL of a sample with a biotinylated monoclonal IL6-specific antibody. This was followed by the second incubation with IL6-specific antibodies labeled with ruthenium complex, and streptavidin-coated microparticles were added. Altogether, both antibodies form a sandwich complex with the antigen of the sample. Next, the mixture was aspirated into the measuring cell, where the microparticles were magnetically captured on the surface of the electrode. The unbound substances were then removed with Procell/ProCell M. The next step involved applying the voltage to the electrode to induce chemiluminescent emission, measured by a photomultiplier. Finally, the results were determined by an instrument-specific calibration curve and generated by a 2-point calibration and a master curve (24).

CRP concentration in serum was measured using an *in vitro* immunoturbidimetric assay (Tina-quant C-reactive protein IV) (25). It is a latex particle-enhanced assay that consists of TRIS buffer, bovine serum albumin (BSA), and latex particles coated with mouse anti-CRP glycine buffer and mouse immunoglobulins. The human CRP was agglutinated with latex particles coated with anti-CRP monoclonal antibodies, and the precipitate was measured by turbidimetry.

### Blood carbamylation

**FIGURE 1:**
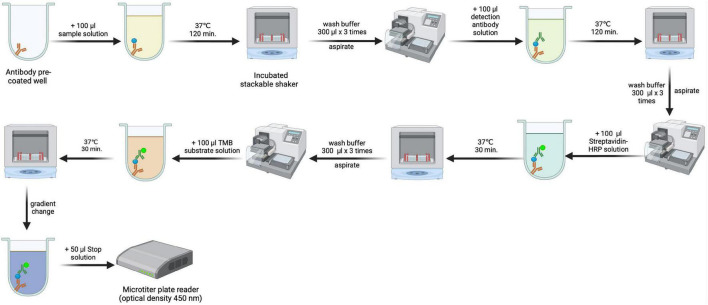
Methodological scheme for estimating serum carbamyl-lysine (CBL) concentration by enzyme-linked immunosorbent assay (ELISA). Created using biorender.com.

The concentration of CBL in serum samples was measured using an enzyme-linked immunosorbent assay (ELISA) obtained from Novatein Biosciences (catalog number BG-HUM09021; Woburn MA 01801 United States) (26). In this case, ELISA measured CBL levels by binding anti-CBL antibodies to the target protein, followed by detection using an enzyme-conjugated secondary antibody that produced a calorimetric signal, recorded by the microplate reader at 450 nm. The procedure for measuring carbamylated protein levels is presented in Figure 1. The sensitivity or minimum detectable dose (MDD) of CBL was determined to be 0.51 ng/ml. Inter-assay reproducibility is <9.6%, and intra-assay reproducibility is <7.1%.

### Statistical analyses

Statistical analysis was performed using GraphPad Prism 9.0 software (GraphPad Software, La Jolla, USA), and the level of statistical significance was set at *p* < 0.05. Normality of distribution was assessed using the Shapiro-Wilk test, and differences between the two independent groups were evaluated using the Mann-Whitney U test. Results are presented in box-and-whisker plots as median (minimum-maximum). The relationship between blood inflammation and protein carbamylation was assessed using Spearman rank correlation. Receiver Operating Characteristic (ROC) analysis was used to assess the diagnostic utility of serum CBL. Area under the curve (AUC) and optimal cut-off values were determined, ensuring high sensitivity and specificity.

The number of subjects was based *a priori* on a pilot study with 12 patients (*ClinCalc* online software). The variables used to calculate the sample size were PCT and IL-6 concentrations. The significance level was set at 0.05, and the power of the test was set at 0.8. The minimum number of patients per group was 23; therefore, 25 survivors and 25 deceased patients were included in the study.

## Results

### Clinical characteristics

Table 1 shows the clinical characteristics of the patients. Importantly, no patients in either group had acute or chronic renal failure.

**TABLE 1:** Clinical characteristics of COVID-19 survivors and COVID-19 deceased patients.

	COVID-19 survivors		COVID-19 deceased patients		*P*-value (deceased vs. survivors)
Sex (male/female)	15/10		15/10		>0.9999
Age (median, min-max)	65 (28–81)		65 (28–81)		>0.9999
**Blood morphology**					
	**Admission**	**Discharge**	**Admission**	**Discharge**	
WBC	5.75 ± 2.64	8.22 ± 7.50	9.24 ± 4.82	17.72 ± 10.61	Admission: <0.0023 Discharge: <0.0001
RBC	4.16 ± 0.77	4.28 ± 6.76	4.45 ± 0.73	4.13 ± 9.19	Admission: 0.2181 Discharge: 0.5159
HGB	13.05 ± 1.76	13.19 ± 1.72	13.6 ± 2.28	12.08 ± 2.92	Admission: 0.3761 Discharge: 0.1085
HCT	37.82 ± 4.91	38.50 ± 4.54	39.51 ± 6.74	36.65 ± 8.16	Admission: 0.4156 Discharge: 0.2369
MCHC	34.51 ± 0.93	34.32 ± 1.40	34.45 ± 1.13	32.85 ± 1.40	Admission: 0.9272 Discharge: 0.001
PLT	172.3 ± 65.23	187.00 ± 63.78	219.6 ± 102.6	240.90 ± 114.30	Admission: 0.0741 Discharge: 0.05
**Comorbidities n (%)**					
Obesity	6 (24)		7 (28)		>0.9999
Hypertension	14 (56)		19 (76)		0.2321
Acute kidney disease	0 (0)		(0)		>0.9999
Chronic kidney disease	0 (0)		(0)		>0.9999
**Clinical status n (%)**					
Pneumonia	4 (16)		14 (56)		0.0072
Invasive ventilation	3 (12)		22 (88)		<0.0001
Hospitalization time	15 ± 3 days		14 ± 7 days		>0.9999

HCT, hematocrit; HGB, hemoglobin; MCHC, mean corpuscular hemoglobin concentration; PLT, platelets; RBC, red blood cells; WBC, white blood cells.

### Blood inflammation

**FIGURE 2:**
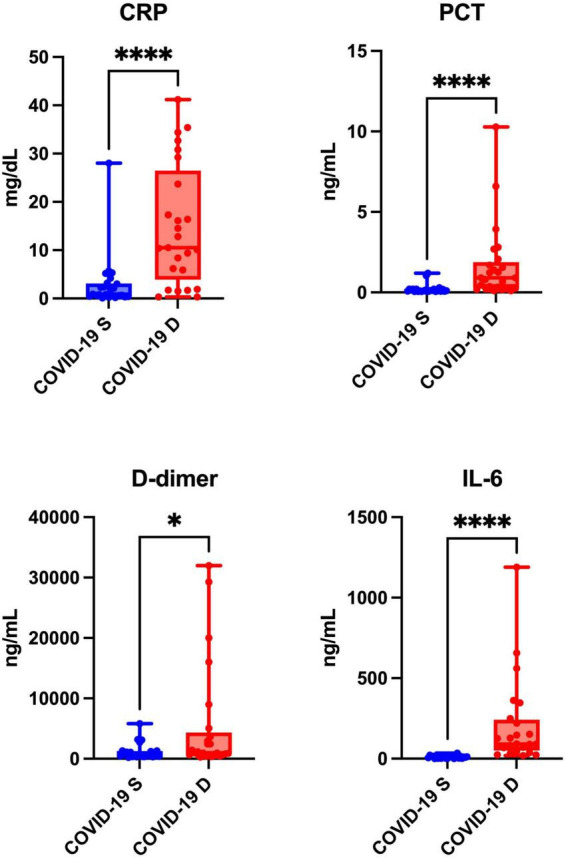
Blood inflammation of COVID-19 survivors (COVID-19 S) and COVID-19 deceased (COVID-19 D) patients. The results are presented in box-and-whisker plots as median (minimum-maximum) and percentiles with individual data points. w, C-reactive protein; PCT, procalcitonin; IL-6, interleukin-6. Differences statistically significant at *<0.05, ****<0.0001.

The levels of inflammatory biomarkers such as CRP (*p* < 0.0001), PCT (*p* < 0.0001), D-dimer (*p* = 0.0214), and IL-6 (*p* < 0.0001) were significantly higher in patients who died of COVID-19 compared to the recovered patients (Figure 2).

### Blood carbamylation

**FIGURE 3:**
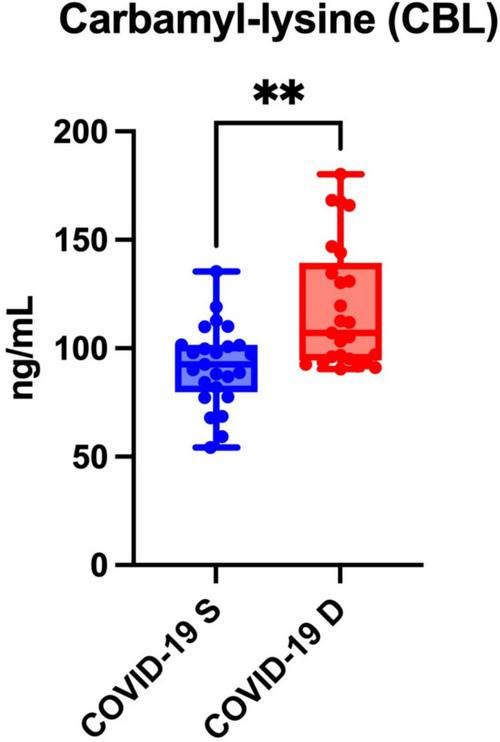
Serum carbamyl-lysine (CBL) of COVID-19 survivors (COVID-19 S) and COVID-19 deceased (COVID-19 D) patients. The results are presented in box-and-whisker plots as median (minimum-maximum) and percentiles with individual data points. COVID-19 S, COVID-19 survived patients; COVID-19 D, COVID-19 died patients. Differences statistically significant at **<0.01.

Serum CBL levels were significantly higher in patients who died from COVID-19 compared to COVID-19 survivors (*p* = 0.0011) (Figure 3).

### ROC analysis

**FIGURE 4:**
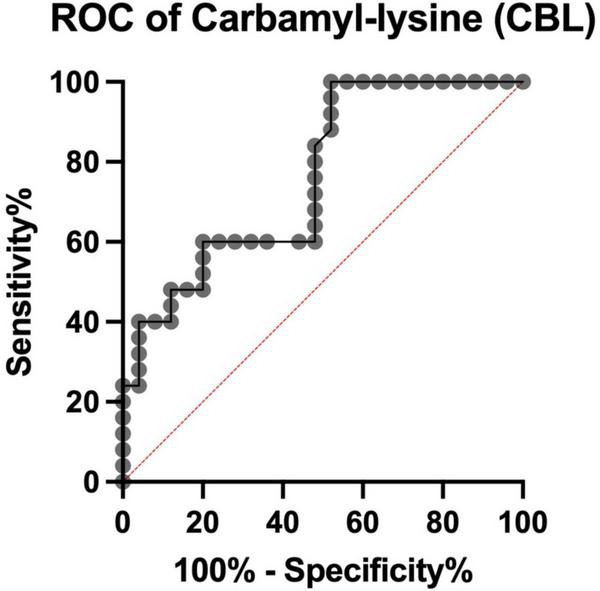
Receiver Operating Characteristic (ROC) curve for serum carbamyl-lysine (CBL) of COVID-19 survivors and COVID-19 deceased patients. ROC, Receiver Operating Characteristic analysis.

We showed that serum CBL levels >101 ng/mL, with a sensitivity of 60% (CI: 40.74 to 76.60%) and specificity of 72% (CI: 52.42 to 85.72%), differentiate COVID-19 deceased from recovered patients. The AUC was 0.76 (CI: 0.63 to 0.89) (Figure 4).

The results of the ROC analysis for inflammatory biomarkers are shown in Table 2.

**TABLE 2:** Receiver Operating Characteristic (ROC) analysis for serum inflammatory biomarkers.

	CRP	PCT	D-dimer	IL-6
AUC	0.8344	0.8976	0.6908	0.99
95% confidence interval	0.7145 to 0.9543	0.8123 to 0.9829	0.5411 to 0.8406	0.9716 to 1.000
*P*-value	<0.0001	<0.0001	0.022	<0.0001
Cut-off	>5.500	>0.3100	>638.0	>19.25
Sensitivity%	76	76	84	95.83
95% CI	56.57%– 88.50%	56.57%–88.50%	65.35%–93.60%	79.76%–99.79%
Specificity%	92	92	58.33	88
95% CI	75.03%–98.58%	75.03%–98.58%	38.83%–75.53%	70.04%–95.83%

AUC, area under the curve.

### Correlations

**FIGURE 5:**
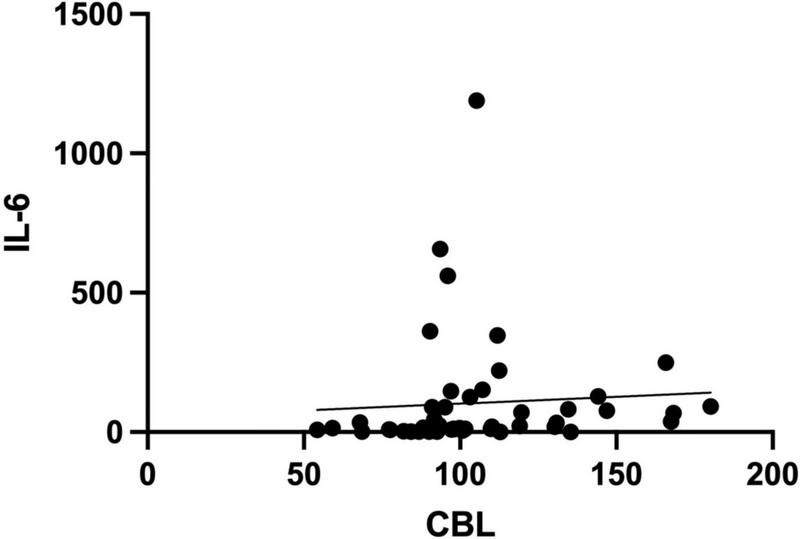
Spearman rank correlation of serum carbamyl-lysine (CBL) and interleukin-6 (IL-6) levels in COVID-19 survivors and COVID-19 deceased patients.

Serum CBL levels were correlated with inflammatory biomarkers such as D-dimer (*r* = 0.38, *p* = 0.008), WBC (*r* = 0.31, *p* = 0.031) and IL-6 (*r* = 0.38, *p* = 0.008) (Figure 5).

## Discussion

Our study is the first to demonstrate increased protein carbamylation in patients with COVID-19. We found that serum CBL levels were significantly higher in those who died of COVID-19 and correlated with inflammatory biomarkers.

**FIGURE 6:**
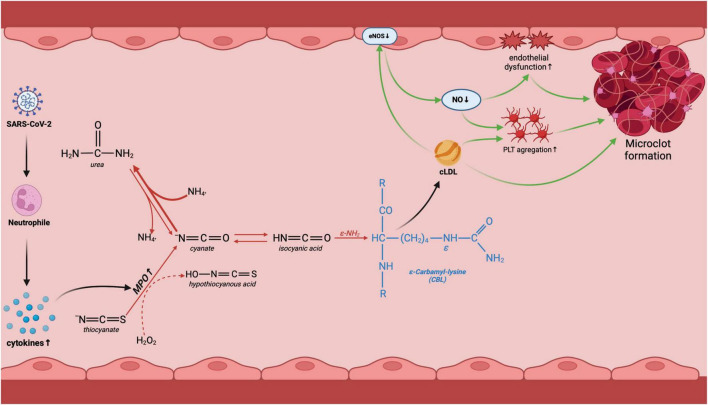
Potential involvement of protein carbamylation in COVID-19-related complications. Following COVID-19 infection, the organism initiates the innate immune response involving neutrophils. This triggers an increase in MPO activity, an enzyme responsible for urea-independent carbamylation. Lysine carbamylation leads to the creation of carbamyl-lysine (CBL), which in turn results in the carbamylation of LDL. Carbamylated low-density lipoproteins (LDL) decrease endothelial nitric oxide synthase (eNOS) activity, causing endothelial dysfunction and platelet aggregation, ultimately leading to the formation of microclots and vascular complications of COVID-19. cLDL, carbamylated low-density lipoprotein; eNOS, endothelial nitric oxide synthase; NO, nitric oxide; MPO, myeloperoxidase; PLT, platelets. Created using biorender.com.

Carbamylation is the non-enzymatic binding of a carbamoyl moiety (−CONH_2_) to the nucleophilic amino groups of proteins. Lysyl residues are particularly vulnerable to carbamylate due to an ε-amino group in the side chain and easy availability to carbamylating agents (16). The main compound responsible for protein carbamylation is electrophilic isocyanic acid. Isocyanic acid is produced from cyanate, formed from urea (27). Not surprisingly, increased carbamylation of proteins is observed in patients with renal failure who have accumulated uremic toxins (19, 20). However, isocyanic acid can also be formed from thiocyanate (SCN^–^) (16, 27). MPO catalyzes the oxidation of thiocyanate to hypothiocyanous acid (HOSCN) in the presence of hydrogen peroxide. Further transformations result in the formation of cyanate and isocyanic acid (16, 27) (Figure 6). Since our study did not include patients with renal failure, it can be assumed that inflammation is the source of increased protein carbamylation in the COVID-19 deceased. It is well known that COVID-19 is associated with an excessive immune system response (28, 29). Our earlier study showed that an increase in cytokine levels in early-stage COVID-19 can be an important diagnostic factor, especially in patients with mild disease symptoms (30). Serum IL-1α, granulocyte-macrophage colony-stimulating factor (GM-CSF), basic fibroblast growth factor (basic-FGF), and monokine induced by interferon-γ (MIG) have high diagnostic potential. They can be used to differentiate COVID-19 severity (30, 31). Another study indicates that increased neutrophil MPO activation and endothelial glycocalyx damage are independently associated with COVID-19 severity (32). MPO levels, activity, and soluble endothelial glycocalyx levels are significantly elevated in patients with COVID-19, increasing proportionally to disease severity. Despite clinical recovery, biomarker levels remain significantly higher. There is also a tendency to increase MPO activity in the plasma of convalescents in both severe and non-severe groups (32). Under conditions of prolonged MPO activation, there may be enhanced isocyanic acid production (33), which increases protein carbamylation. Unfortunately, in our study, we did not isolate neutrophils from whole blood, and thus did not assess MPO activity. A potential link between protein carbamylation and inflammation may be evidenced by the positive correlation of serum CBL with IL-6, D-dimer, and WBC. It is well known that IL-6 induces platelet activation, causing endothelial damage and thrombotic disorders with increased D-dimer production (34). Carbamylated proteins can also induce endothelial dysfunction through ROS overproduction and activation of lectin-like oxidized low-density lipoprotein receptor-1 (LOX-1) by uncoupling endothelial nitric oxide synthase (eNOS) (35–38) (Figure 6).

Protein carbamylation can inhibit many biologically active enzymes or hormones (39). Carbamylated proteins aggregate and accumulate in tissues, are more resistant to proteolysis, and undergo further modification by oxidation or glycation (16, 39). Protein carbamylation is also a biomarker of molecular aging, making it associated with many pathological conditions (40). Indeed, the involvement of protein carbamylation has been described in chronic kidney disease (41), rheumatoid arthritis (42), cataracts (43), atherosclerosis (44), and neurological disorders (45). It was shown that carbamylation of proteins can be induced by neutrophil extracellular traps (NETs) (46). Since COVID-19 causes severe inflammatory reactions with the formation of NETs (47, 48), neutrophils may be the cause of the increased CBL levels. However, studies on the diagnostic utility of carbamylation biomarkers in COVID-19 patients are lacking. Our ROC analysis showed that the AUC for serum CBL differentiating the deceased and recovered patients on COVID-19 was 0.76. However, it should be noted that the blood samples were taken during the last blood measurement before hospital discharge for survivors or the last blood measurement before death for patients who died from COVID-19. Since no measurements were taken upon admission to the hospital, the baseline carbamylation predictive value cannot be used as a biomarker of the worst outcome. Further studies should evaluate the usefulness of CBL in monitoring disease progression. The sensitivity (60%) and specificity (72%) of CBL are moderate and lower than those of routinely used inflammatory biomarkers, which does not indicate a high diagnostic potential. Although the sample size has been statistically determined, evaluation of the diagnostic potential of CBL is also required on a larger patient population. In addition, the severity of protein carbamylation should be analyzed concerning the patient’s clinical condition [e.g., using the Modified Early Warning Score (MEWS)], lifestyle factors (e.g., diet, smoking, physical activity), concomitant diseases, and medications taken (49). Future studies should also assess other biomarkers of protein carbamylation, e.g., carbamylated hemoglobin, carbamylated albumin, and carbamylated low-density lipoproteins (LDL).

## Conclusion

In conclusion, COVID-19 is associated with excessive protein carbamylation. Evaluation of serum CBL levels with moderate sensitivity and specificity differentiates COVID-19 deceased from recovered patients. Inflammation may be a source of increased protein carbamylation in COVID-19. A thorough understanding of the consequences of increased CBL production may clarify the consequences of COVID-19 complications. Therefore, our research indicates the need for further studies.

## Data Availability

The raw data supporting the conclusions of this article will be made available by the authors, without undue reservation.
